# Optic Fiber Microsensor Reveals Specific Spatiotemporal Oxygen Uptake Profiles at the Mammalian Ocular Surface

**DOI:** 10.3390/bios13020245

**Published:** 2023-02-09

**Authors:** Qin Sun, Li Ma, Fernando Ferreira, Chelsea Brown, Brian Reid, Min Zhao

**Affiliations:** 1 Department of Dermatology, Institute for Regenerative Cures, School of Medicine, University of California, Davis, CA 95816, USA; 2 School of Life Science, Yunnan Normal University, Kunming 650092, China; 3 Departamento de Biologia, Centro de Biologia Molecular e Ambiental (CMBA), Universidade do Minho, 4710-057 Braga, Portugal; 4 Department of Ophthalmology & Vision Science, Institute for Regenerative Cures, School of Medicine, University of California, Davis, CA 95816, USA

**Keywords:** cornea, oxygen uptake, spatiotemporal profile, self-referencing probe, oxygen sensor, optical fiber sensor

## Abstract

Oxygen (O_2_) uptake by cells and tissues is a critical indicator of metabolic demand, changes in microenvironment, and pathophysiology. O_2_ uptake from the atmosphere accounts for virtually all the O_2_ consumption in the avascular cornea; however, a detailed spatiotemporal profile of corneal O_2_ uptake (COU) remains undetermined. Here, we used a non-invasive self-referencing optical fiber O_2_ sensor—the scanning micro-optrode technique (SMOT)—to report the O_2_ partial pressure and flux variations at the ocular surface of rodents and non-human primates. In vivo spatial mapping in mice revealed a distinct COU, characterized by a centripetal gradient with a significantly higher O_2_ influx at the limbus and conjunctiva regions than at the center of the cornea. This regional COU profile was reproduced ex vivo in freshly enucleated eyes. The centripetal gradient was conserved across the following species analyzed: mice, rats, and rhesus monkeys. In vivo temporal mapping in mice showed a significant increase in the O_2_ flux in the limbus in the evening compared to other times. Altogether, the data unveiled a conserved centripetal COU profile, which may be associated with the limbal epithelial stem cells residing at the intersection of the limbus and conjunctiva. These physiological observations will serve as a useful baseline for comparative studies with contact lens wear, ocular disease, diabetes, etc. Moreover, the sensor may be applied to understand the responses of the cornea and other tissues to various insults, drugs, or changes in the environment.

## 1. Introduction

Oxygen (O_2_) is an essential molecule of metazoan life [1]. It acts as the final acceptor of the electron transport chain, is an integral cofactor in enzymatic reactions, and plays a key role in signal transduction pathways [2,3]. Understanding the O_2_ consumption of different cells, tissues, and discrete structures can give us deeper insights into their metabolic demand and physiology [4]. Sites of high O_2_ intake are indicative of high metabolic demand [5]. Variation in O_2_ levels is also a characteristic of distinct cellular microenvironments. For example, recent studies have found that changes in O_2_ flux gradients can be indicative of stem cell and progenitor niches [6]. In most tissues, the vascular system meets the O_2_ demands, but that is not the case for the cornea, which is essentially an avascular tissue [5]. Corneal O_2_ is sourced through atmospheric passive diffusion, except for a peripheral zone with a negligible contribution from nearby vasculature [2,7,8]. Since the cornea acts as a barrier between the eye and the environment, it must continually cull and renew itself to maintain its integrity [9,10]. This rapid tissue renewal is also essential to maintain its transparency to support the sensory organ in light refraction [9]. These functions are metabolically taxing and steadily demand O_2_ [10]. Proper oxygenation is also necessary to quell hypoxic swelling, edema, epithelial and stromal thinning, acidosis, epithelial microcysts, and hypoesthesia [11]. Consequently, the cornea is a highly metabolically active tissue and therefore must uptake adequate levels of O_2_ to meet the high demand [4,10].

Corneal oxygen uptake (COU) rates can be measured to monitor the O_2_ demands of the tissue [12]. These measurements may give insight into subpopulations of cells such as limbal epithelial stem cells (LSCs), whose location has been a source of some debate. Moreover, COU measurements may serve as a baseline to compare against disease, wound healing, and contact lens wear [5].

COU measurements pose a few considerations, one being the location of measurement. A previous study utilized a Hill and Fatt polarographic O_2_ sensor to measure COU rates in human cornea. They found a homogenous rate of uptake across the different measurement sites [12]. However, they measured the center of the cornea and its periphery, which are similar physiologically. They did not measure the limbus or conjunctiva regions, which may have different characteristics based on their distinct anatomical composition. Another consideration is the time of day that the measurements are taken. As with most tissues, the cornea is subject to the influence of the circadian rhythm [13]. The circadian rhythm is an intrinsic biological characteristic that influences normal physiology based on a 24 h cycle [13]. This rhythm is controlled at the molecular level through transcription and translation pathways. One physiological function that the circadian rhythm has considerable control over is metabolism and, consequently, O_2_ consumption [13]. The specific changes noted in the cornea were that it is thickest in the early morning and thinnest in the late evening [13]. It has also been seen that corneal mitosis and DNA synthesis exhibit diurnal changes, both increasing during the day and decreasing at night [9,13]. Similar observations have been seen in the limbus region of the epithelium [13]. Recent studies have confirmed the influence of the circadian clock in the cornea through RNAseq, in which it was found that a quarter of the transcriptome was temporally regulated [13]. These findings taken together point to the possibility of a distinct spatiotemporal O_2_ uptake profile of the cornea.

The overall goal of the present study was to gain a more comprehensive spatiotemporal understanding of the O_2_ intake of the cornea. To meet this goal, we had two main aims: (1) to spatially measure O_2_ uptake at different regions in the cornea (center, periphery, and limbus) and sclera (conjunctiva); and (2) to temporally measure O_2_ uptake at different times of day (morning, midday, and evening) at the cornea and sclera. We also took these measurements in different relevant biomedical animal models (rodents and non-human primates) to understand the putative conserved phenomena.

To measure the partial pressure of O_2_ (*pO*_2_) at the cornea, we used a recently described non-invasive self-referencing optical fiber microsensor (also termed optrode or optode) and converted these measurements to O_2_ flux using Fick’s first law of diffusion [14]. This method, otherwise referred to as the scanning micro-optrode technique (SMOT), uses an O_2_-sensitive fluorophore at the tip of the sensor to quantify O_2_ via fluorescence quenching, since O_2_ is one of the best-known quenchers [15]. This approach, combining an optical O_2_ fiber sensor, self-referencing, computerized control, and online computation, provides on-site measurement, presentation, and visualization of O_2_ concentration and O_2_ fluxes with a high spatiotemporal resolution. Importantly, this optic-based probe (micro-optrode) does not consume O_2_ in the measurement, making the SMOT more accurate and easier-to-use than other methods such as polarographic electrodes [4,16]. Together with the extensive spatiotemporal approach in different biomedical models, we expect that this technique will enable researchers to gain new insights into the O_2_ uptake profile of the cornea and other tissues. This will facilitate a better understanding of the corneal physiology and derive a baseline for future ophthalmological and pathological comparisons.

## 2. Materials and Methods

### 2.1. Animals

**Figure 1:**
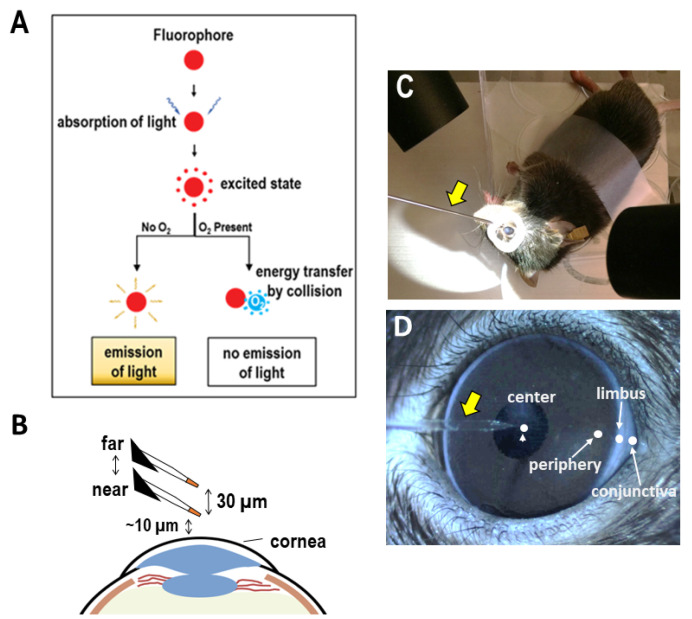
Optrode for measurement of O_2_ uptake in mouse cornea in vivo. (**A**) Principle of the fluorescence-quenching-based O_2_ measurement. O_2_ collision with the fluorophore quenched emission fluorescence and therefore reported O_2_ concentration. (**B**) Spatial resolution was dependent on the diameter of the micro-optrode tip (~20 μm), which was positioned about 10 μm above the region of interest. The moving step between near and far pole was 30 μm (not to scale). (**C**) A hydrophobic well of vacuum grease was built around mouse eyes, and artificial tear solution was filled in the well as measuring medium. Optrode is indicated by the yellow arrow. (**D**) Four measurement positions (center and periphery of cornea, limbus, and bulbar conjunctiva) are indicated. O_2_ uptake therefore could be measured with accurate spatial and temporal information. The yellow arrow shows the micro-optrode.

The purchase of, housing of, and experiments on animals described in this study were approved by the Institutional Animal Care and Use Committee at the University of California, Davis. Eight-week-old male C57BL/6 mice were used in ex vivo and in vivo measurements. All mice were housed in a vivarium with controlled temperature and humidity and with regular dark/light shifts. Prior to the experiments, mice were anesthetized with a mixture of 10 mg mL^−1^ ketamine (Zetamine, ketamine hydrochloride injection, VetOne) and 0.1 mg mL^−1^ dexmedetomidine (Dexdomitor, Zoetis, Parsippany, NJ, USA). After anesthetization, mice were immobilized on a plastic plate by tape. A ring well was made using hydrophobic grease (high-vacuum silicone grease, Dow Corning, Midland, MI, USA) around the eye for measurement and was filled with a few drops of BSS+ buffer (Alcon Laboratory, Inc., Fort Worth, TX, USA) as the measurement medium, as shown in Figure 1C.

Freshly isolated eyes were obtained from male C57BL/6 mice, male Wistar rats (*Rattus norvegicus*) euthanized at 10 weeks of age, and male and female rhesus monkeys (*Macaca mulatta*) culled humanly for other experiments (all under 4 years old) from the California National Primate Research Center. Excised eyes were mounted in custom-made chambers which fit their sizes for measurement.

### 2.2. Scanning Micro-Optrode Technique (SMOT)

The SMOT is mainly composed of a micro-optrode, an amplifier and a computerized motion control. The detailed technical specification and our protocols have been published recently [3,14]. O_2_ was measured non-invasively with an O_2_-selective optrode (optical electrode), as detailed in our previous publications [3,14]. Pulled optical fiber with a solid-state O_2_-sensitive fluorophore coated on its tip formed the probe. O_2_ quenched the fluorophore and affected the emission following excitation with blue–green light (λ = 505 nm) from an LED source. The changes in the emission thus quantitatively indicated O_2_ concentrations, as shown in Figure 1A. This was possible because the relationship between the phase angle and concentration of O_2_ is virtually linear at physiologically relevant levels (*pO*_2_ 0–32%) [14]. Ready-to-use needle-type housing optrodes (probes) (PreSens, NTH-PSt1-L5-TS-NS40) were incorporated into a turn-key system scanning micro-optrode technique (SMOT; Applicable Electronics, New Haven, CT, USA). SMOT and ASET (automated scanning electrode technique) interface software (version LV4) were obtained from Applicable Electronics, LLC, and Science Wares, Inc., Falmouth, MA, USA.

Calibration was conducted as previously described [3,14]. Briefly, a 2 M sodium bisulfite (mixture of NaHSO_3_ and Na_2_S_2_O_5_, Sigma-Aldrich, St. Louis, MI, USA) solution and air-pumped deionized water were used as calibration solutions for 20 min, for determined partial oxygen pressures (*pO*_2_) of 0% (anoxia) and 20.95% (environmental normoxia), respectively [14]. The tip of the optrode was immersed into calibration solutions for a few minutes until the readings fluctuated in a narrow range, after which the *pO*_2_ levels were recorded and used for calibration.

During measurements, the ASET software computed the *pO*_2_ (%) in real time using the Stern–Volmer mathematical formulation for fluorescence quenching [14,17]. Then, this *pO*_2_ (%) value was plugged into the following equation [14,18] to calculate the local O_2_ concentration:

(1) $$\left[O_{2}\right]\;\left(\mu M\right)=\frac{p_{atm-p_{W}\left(T\right)}}{p_{N}}\times \frac{\frac{pO_{2}}{0.2095}}{100}\times 0.2095\times \alpha \left(T\right)\times 1000\times \frac{1}{V_{M}}$$

where *patm* is the local atmospheric pressure, *pW(T)* is the vapor pressure of water (26.507 mbar at 22 °C (mean room temperature)), *pN* is the standard atmospheric pressure (1013.25 mbar at sea level), $\frac{\frac{pO_{2}}{0.2095}}{100}$ is the ratio of O_2_ in the gas mixture (oxygen and nitrogen relative to the total air), *α(T)* is the Bunsen absorption coefficient (29.908 cm^3^ (O_2_) cm^−3^ at 22 °C), and *V_M_* is the molar volume (22.414 L mol^−1^).

The stepped motion control of the SMOT enabled the measurement of the O_2_ flux according to Fick’s first law of diffusion. The O_2_ flux describes the amount of uptake or outflow of net O_2_ per unit area per unit of time. To achieve this, the optrode was positioned under a microscope at ~10 µm from the ocular surface (near position). An excursion of 30 µm away from the ocular surface was set, which was controlled by the SMOT system to move between the far position (far) and the near position (near) at a frequency of ~0.1 Hz (11 s per iteration), as shown in Figure 1B. Reference values were recorded with the optrode away from the cornea tissue (>>1 mm from the ocular surface). J_O2_ (fluxes of O_2_) levels were recorded for 2–5 min (~10–30 data points) in order to enable a stable signal to be averaged. Measurements were performed at room temperature in the regions and times specified, as shown in Figure 1C,D, apart from the metabolic experiments performed,. Data were acquired and extracted using ASET-LV4 (Science Wares) and were processed and compiled using Excel (Microsoft).

The [*O*_2_] (from Equation (1)) value was converted to pmol cm^−3^ and included in Fick’s first law to calculate the fluxes:

(2) $$J_{O_{2}}\;\left(pmol\;cm^{-2}\;s^{-1}\right)=-D\times \frac{\delta O_{2}}{\delta x}$$

where *D* is the diffusion coefficient of dissolved O_2_ (2.42 × 10^−5^ cm^2^ s^−1^), and *δO*_2_ is the concentration difference (in pmol cm^−3^) over the excursion *δx* (30 μm). The reference value was subtracted.

The SMOT system thus achieved a high resolution for the measurements of O_2_ concentration spatially, i.e., ~20–50 μm, and temporally, i.e., ~2 s, and for the measurement of O_2_ fluxes, the resolutions achieved were ~60–100 μm spatially and ~11–22 s temporally, respectively.

### 2.3. Measurement at Cornea

**Figure 2:**
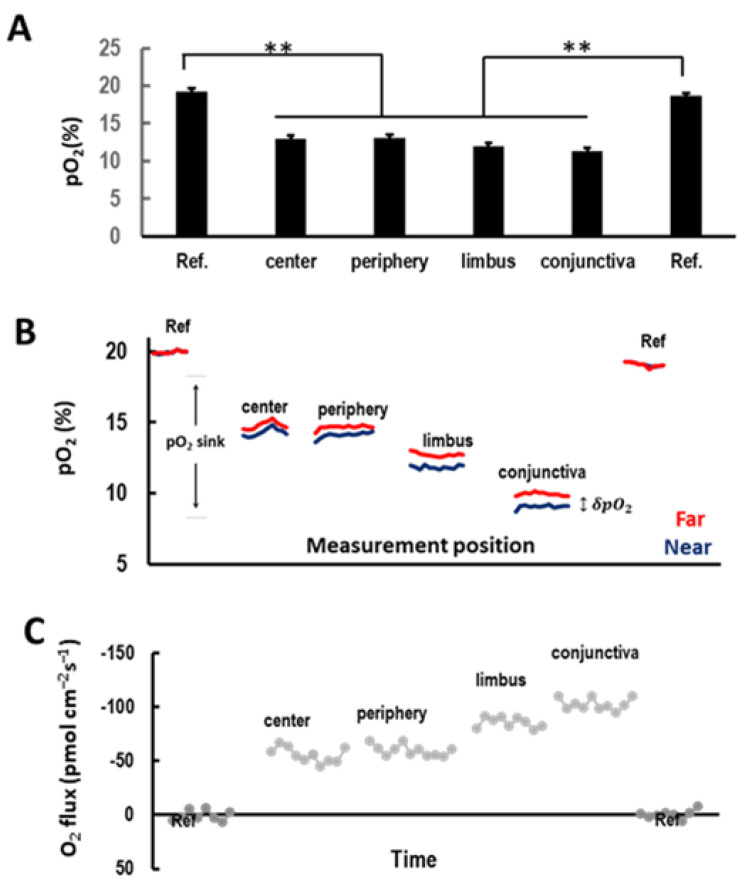
O_2_ concentration and uptake measured by optrode at ocular surface in vivo showed highest O_2_ uptake at the limbus and conjunctiva. (**A**) A characteristic O_2_ concentration measurement in an intact cornea. Ocular surface was an O_2_ sink, where O_2_ was taken up by the cornea compared to reference positions (Ref. in measuring buffer). (** *p* < 0.001) (**B**) The red and blue lines show the partial pressure of O_2_ (*pO*_2_) concentration measured in far and near points at 30 μm distance, as shown in Figure 1B ~10 μm from ocular surface. (**C**) O_2_ fluxes calculated using the O_2_ concentration difference (δ*pO*_2_) based on Fick’s law. Negative values indicate that the O_2_ flux direction was from the atmosphere into the cornea.

The anesthetized mice with hydrophobic wells around the eye (in vivo) or enucleated eyes in wells (ex vivo) were brought to the measurement stage. A calibrated probe was carefully moved into the measuring medium under the camera and, using a 3D micro-positioner, the micro-optrode was manually moved into the measuring buffer solution in the same view of the region of measurement. The motion controller and ASET software were used to finely move the tip of the optrode to the same focal plane as the region of measurement at about 10 μm perpendicular from the surface. The moving step of the optrode was set to 30 μm between the near and far measuring points, as shown in Figure 1B. The probe in the measurement position was allowed to stabilize for a few minutes, and then reference readings were collected in the measuring buffer more than 1 mm away from specimens for about 2 min, as shown in Figure 2. The probe was then moved back to the measurement position to begin recording data. After measurements were finished, reference readings were taken again to ensure consistency.

### 2.4. Circadian Rhythm Measurements

Three male C57BL/6 mice were used to measure the circadian rhythm of corneal O_2_ uptake. Measurements were taken on the cornea (center, periphery, limbus) and sclera (conjunctiva), as shown in Figure 1D, in the morning (9 am), at midday (1 pm), and in the evening (5 pm).

### 2.5. Statistical Analysis

Data are expressed as mean ± standard error of the mean (S.E.M.). Differences between groups were compared using the parametric Student t-test, and statistical significance was considered if the *p*-value < 0.05. All data analysis, graphs, and statistical calculations were conducted using Microsoft Office Excel.

## 3. Results

### 3.1. Ocular Surface Uptakes Oxygen in a Centripetal Gradient In Vivo

**Figure 3:**
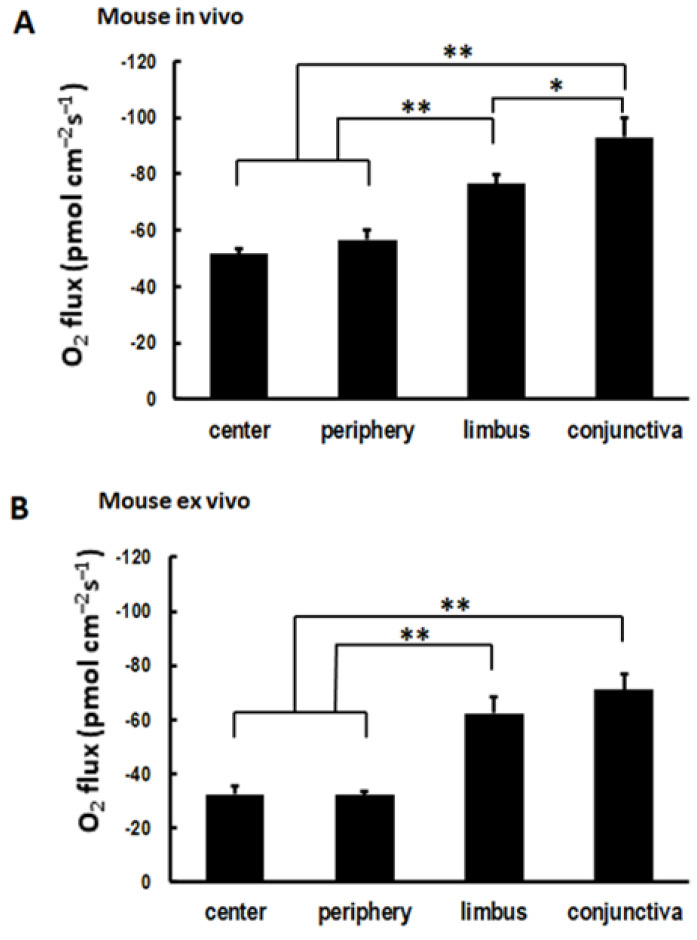
Cornea of enucleated eyes maintained the centripetal gradient. (**A**) O_2_ uptake across the cornea measured in vivo in mice showed centripetal uptake pattern. (**B**) O_2_ uptake across the cornea measured ex vivo in mice showed centripetal uptake pattern (*: *p* < 0.05; **: *p* < 0.01). Data are expressed as mean ± S.E.M. from eight eyes in four mice.

To seek the putative regional differences in COU across the ocular surface of in vivo mice, we mapped the *pO*_2_ levels with a micro-optrode. After anesthesia of the mice, we positioned the probe in the center, periphery, and limbus of the cornea and in the conjunctiva of the sclera, as shown in Figure 1C,D. When the probe was positioned more than 1 mm away from the ocular surface, the *pO*_2_ of readings were ~20%, which indicated the dissolved O_2_ saturation in the measuring BSS+ buffer, as shown in Figure 2A. When the probe was brought to the near position ~10 μm from the ocular surface, the *pO*_2_ values dropped significantly, as shown in Figure 2A,B. The *pO*_2_ levels decreased to ~75% of the saturation level at the center and periphery of the cornea, ~65% at the limbus, and ~55% conjunctiva, all significantly lower than the *pO*_2_ level at the reference position (>1 mm away from the ocular surface), as shown in Figure 2A,B. The difference between the *pO*_2_ values during the excursion of the probe (‘near’ and ‘far’ positions; Figure 1B) provided the O_2_ concentration difference (δ*pO*_2_), as shown in Figure 2B, that was used to compute the O_2_ flux following Fick’s first law of diffusion, as shown in Figure 2C (see Section 2 for details). Thus, the ocular surface presented a sustained sink of O_2_, following a centripetal gradient of O_2_ uptake. The uptake of O_2_ of the limbus and conjunctiva was substantially higher than that of the center and periphery of the cornea, as shown in Figure 2A–C. Negative values were indicative of O_2_ influx, i.e., the corneal O_2_ uptake. The calculated COU showed a striking centripetal gradient, with the highest uptake being at the outer edges (limbus and conjunctiva) and the lowest being at the center of the ocular surface, as shown in Figure 2C and Figure 3A.

### 3.2. Cornea of Enucleated Eyes Maintains the Centripetal Gradient

**Figure 4:**
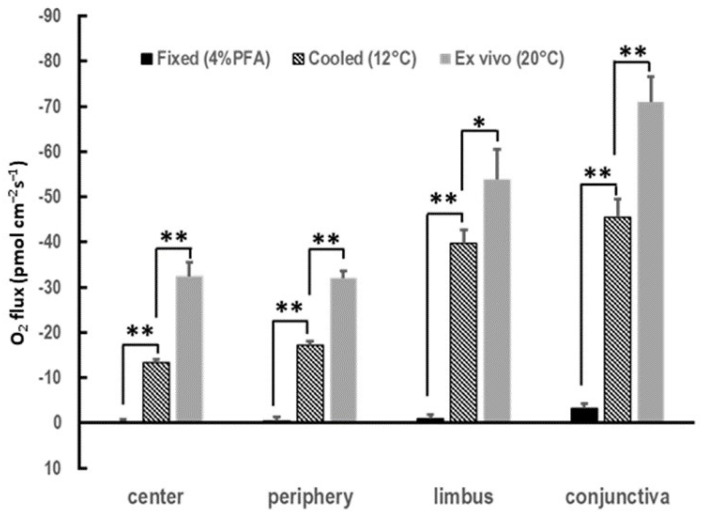
O_2_ Flux values resulted from active metabolism of the tissue. Ex vivo mouse eyes were subject to cooling and fixation to verify that O_2_ measurements were a result of active metabolism of the tissue. Ex vivo eyes measured at 20 °C were used as the baseline, while eyes cooled to 12 °C showed significantly less O_2_ flux, and fixed eyes showed virtually zero O_2_ flux. Data are expressed as mean ± S.E.M. from eight eyes in four mice (*: *p* < 0.05; **: *p* < 0.01).

Next, we asked whether the spatial profile of the COU in vivo could be reproduced ex vivo in the cornea, since ex vivo corneal cultures and mouse eyeball cultures are used frequently in the research of ocular surface biology. This reproduction is important as it will expedite experiments with large animals and when in vivo measurements are unfeasible or impractical. We observed a consistent centripetal gradient in the COU ex vivo, with significantly higher uptake in the limbus and conjunctiva than at the center and periphery of the cornea (Figure 3B). This spatial profile was similar to that observed in vivo, as shown in Figure 3A. The magnitude of the uptake appeared to be lower in the ex vivo samples than in the in vivo ocular surfaces, as shown in Figure 3A,B. A potential explanation could reside in the lower temperature during the ex vivo measurements since the data were acquired at room temperature (~20 °C) rather than at body temperature when the measurements were made in vivo. Indeed, when we lowered the bathing solution temperature to 12 °C, the COU significantly decreased, as shown in Figure 4. To further verify that the reported COU was due to the activity of metabolizing cells, we chemically fixated the ex vivo eyes using paraformaldehyde (4%, *w*/*v*). This treatment resulted in virtually no detectable COU, as shown in Figure 4.

### 3.3. Spatial COU Profile Is Conserved across Species

**Figure 5:**
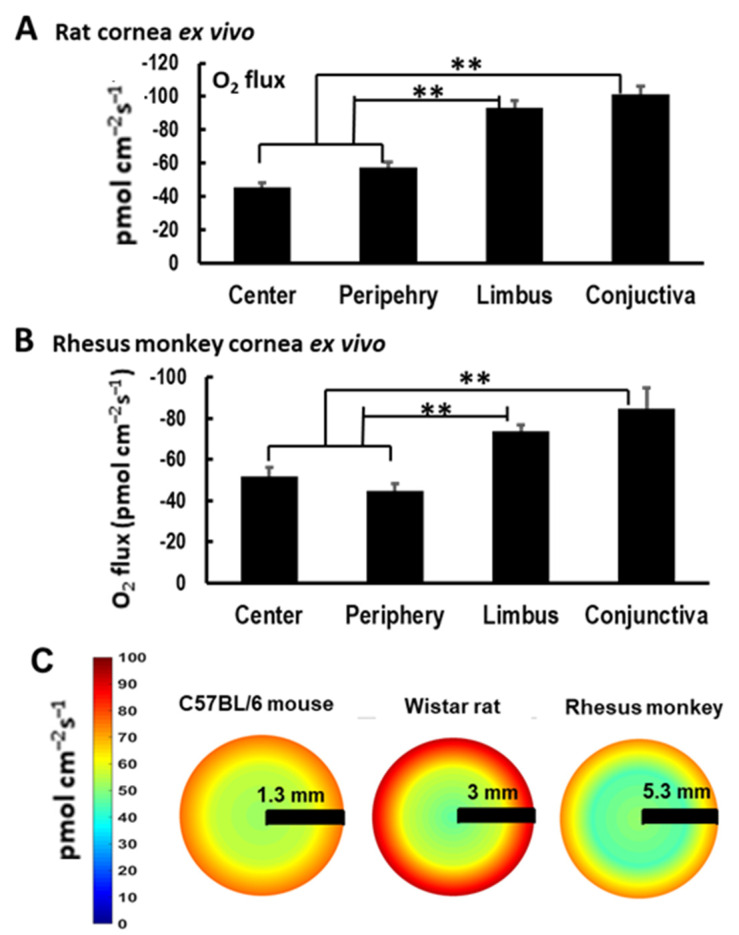
The limbus and conjunctiva had the highest O_2_ uptake across the cornea in rats and rhesus monkeys and showed a conserved centripetal O_2_ uptake gradient. (**A**,**B**) O_2_ uptake across the cornea measured in ex vivo rat and rhesus monkey eyes. Limbus and conjunctiva had a unique and significant higher O_2_ uptake (**: *p* < 0.01). Data are expressed as mean ± S.E.M. (**C**) Pseudo color images show O_2_ uptake across the cornea, showing conserved peak O_2_ uptake at the limbus and conjunctiva with a centripetal gradient.

To gain insights into the evolutionary conservation of the spatial centripetal gradient across the ocular surface, we started by measuring the COU in another rodent, i.e., rats. The ex vivo measurements revealed the same spatial profile with a significantly larger COU at the limbus and conjunctiva when compared to the center and periphery of the cornea, as shown in Figure 5A. Next, we moved to a higher taxon and analyzed the spatial COU in a non-human primate, i.e., rhesus monkeys. Notably, the measured centripetal gradient was reproduced with remarkable accuracy, as shown in Figure 5B. Finally, we plotted the measured COU across the ocular surface using a heatmap. This visualization showed a consistent and reproducible centripetal gradient of O_2_ uptake in the different species analyzed, as shown in Figure 5C. The heatmap also showed that while the profiles were similar, the magnitudes of the uptake were species-specific with larger O_2_ uptakes in the rat and rhesus monkeys than in the mice, which seemed to roughly correlate with the size of the eyeball, as shown in Figure 5C.

Therefore, the COU profile was conserved across the rodents and the non-human primates, suggesting an evolutionary conservation that may apply to other mammals.

### 3.4. O_2_ Uptake Increases in the Limbus in the Evening

**Figure 6:**
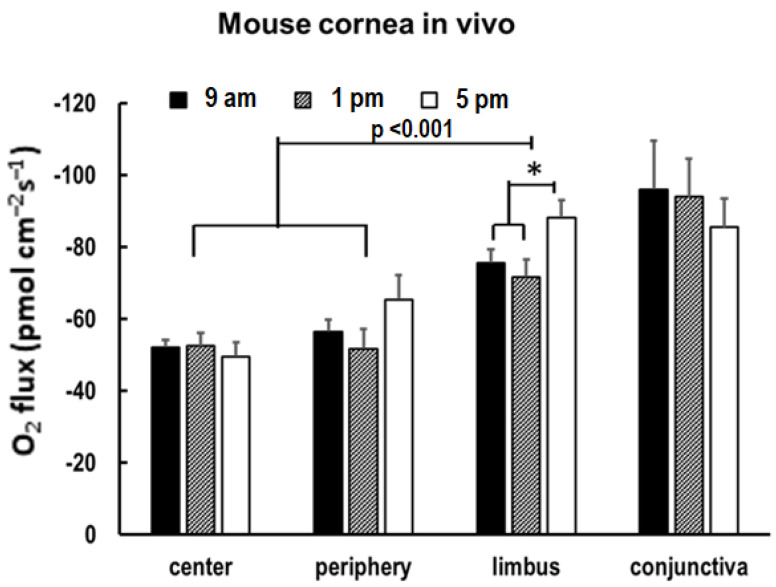
O_2_ uptake at the limbus measured in in vivo cornea showed a significant increase (*: *p* < 0.05) compared to the measurements at 9 am and 1 pm. Data are expressed as mean ± S.E.M. from six eyes in three mice.

To test whether the COU varies temporally during the time of day, we measured the O_2_ uptake in the in vivo mouse eyes in the morning (9 am), at midday (1 pm), and in the evening (5 pm). We observed a similar significant centripetal COU gradient in the three temporal periods, as shown in Figure 6. When comparing the individual regions, we found no significant differences along the time of day at the center of the cornea and at the conjunctiva, as shown in Figure 6. Intriguingly, the limbus O_2_ uptake shifted significantly in the evening, with a ~20% increase in the COU when compared to the morning and midday levels with statistical significance, as shown in Figure 6. The O_2_ uptake at the periphery appeared to increase in the evening, as shown in Figure 6, but did not reach statistical significance.

## 4. Discussion

In this study, we used an optical fiber sensor to examine the O_2_ uptake at the ocular surface of in vivo mice and ex vivo mice, rats, and rhesus monkeys. We generated a spatiotemporal COU profile at the ocular surface, which was conserved across the mammalian species evaluated (rodents and non-human primates). Spatially, we found a centripetal gradient of O_2_ influx, with the highest uptake at the limbus and conjunctiva, which decreased towards the center of the cornea. The O_2_ uptake was absent in the fixed tissues and decreased in the eyes kept at low temperatures, which confirmed that the measurements were associated with the active metabolism and physiology of the tissues. Notably, the limbus O_2_ uptake fluctuated in the day, increasing significantly in the evening, whereas the O_2_ uptake at the other ocular surface regions remained unchanged.

Since O_2_ uptake is a well-established indicator of metabolic activity, we can confidently assert that the limbus and conjunctiva had higher metabolic rates than the more central regions of the cornea. This was likely due to the limbal epithelial stem cells (LSCs) that are believed to reside at the intersection of the limbus and conjunctiva and the transiently amplifying cells that are used to replenish the epithelial cells [19]. Since the cornea is in a state of constant renewal as it interfaces with the outer atmospheric environment, its stem cell population is constantly working to maintain the health of the cornea [19]. This turnover would lead to high levels of O_2_ uptake, as was observed in the present study. These observations will serve as a useful baseline and framework for comparative studies with, for instance, contact lens use, ocular disease, and diabetes.

Temporally, we found that in the mouse model, there was a substantial shift only in the limbus region. We observed a significant increase in the O_2_ influx at 5 pm. The reason for this uptick is not yet clear, but it may point to an upregulation in LSC activity in the evening. It has been seen that the murine epithelial thickness increases significantly in the evening as they become more active, peaking around 10 pm [13]. It is possible that an upregulation in LSC activity would eventually lead to epithelial thickening, since desquamification has been seen to occur in the early morning [13]. However, the fact that the limbus was the only region with a significant difference was surprising, since there is a large body of evidence that shows that the cornea is temporally regulated. In mice, the cornea center and periphery have been observed to have upregulated cell proliferation in the first half of the day, persisting from 4 am to 2 pm [13]. We may not have observed this phenomenon due to the experimental procedures disrupting the eye’s normal physiological processes (exposure to light and dark straying from normal schedules). Additionally, we may not have encompassed physiologically relevant time points in our experimental design. Further experiments should be performed either studying O_2_ uptake in mice encompassing nighttime and early morning measurements and/or utilizing a diurnal model closer to ourselves, such as a non-human primate model.

This study also confirmed the micro-optrode-based SMOT system as a reliable and easily applicable technique for measuring O_2_ uptake with high spatial and temporal resolutions at the ocular surface. Early researchers studied O_2_ reduction using platinum and gold electrodes, but these were not reliable sensors due to artifacts [20]. Later, Clark developed a micro-electrode by covering a cathode with a resistive membrane, which minimized the artifacts but lost sensitivity for O_2_ consumption due to its smaller sensor [21,22,23]. The SMOT technique is based on the effect of dynamic luminescence quenching by collisions with molecular O_2_ that is not consumed during measurement. Combined with its self-referencing capability, O_2_ fluxes can be reliably and accurately measured at the ocular surface non-invasively and in vivo [24], thus offering an optical fiber sensor system for many other tissues where O_2_ consumption is important. This non-invasiveness and high sensitivity make the SMOT system well suited for experimental and clinical applications and therefore may well be a good standard for O_2_ flux measurements.

## Data Availability

Data is contained within the article or supplementary material.
